# Unequal access to restorative coastal blue–green places: social vulnerability and place-based well-being opportunities in a coastal metropolis

**DOI:** 10.3389/fpubh.2026.1917150

**Published:** 2026-10-07

**Authors:** Baijing Liu, Xinping Yu, Lu Yu, Hongzhen Zheng, Guannan Li

**Affiliations:** 1 School of Resources and Environmental Engineering, Ludong University, Yantai, Shandong, China; 2 Jinan Land Reserve Center, Jinan, Shandong, China; 3 School of Water Conservancy and Environment, University of Jinan, Jinan, Shandong, China; 4 Zhanhua District Natural Resources Bureau of Binzhou City, Binzhou, Shandong, China; 5 Ulsan Ship and Ocean College, Ludong University, Yantai, Shandong, China

**Keywords:** blue–green exposure, coastal metropolis, environmental justice, place-based well-being, social vulnerability

## Abstract

**Background:**

Urban blue–green environments are vital components of the urban exposome, shaping daily opportunities for physical activity, stress mitigation, and broader public health. However, these benefits depend on daily accessibility. In compact coastal metropolises, extensive nature may coexist with highly unequal residential access. This study investigates whether socially vulnerable residential areas in Shenzhen experience reduced exposure to public green and coastal blue spaces.

**Methods:**

Using 4,071 residential patches, a residential-scale exposure framework was developed. Social vulnerability was estimated with housing price, property-management fee, dwelling age, and neighborhood commercial-service structure. Blue–green exposure was calculated through a supply–demand model integrating walking-network accessibility, 15-min living-circle coverage, and residential demand. Lorenz curves evaluated capacity-weighted inequality. Scatterplots and quadrant typology analysis identified nonlinear associations and areas where high vulnerability coincided with low exposure.

**Results:**

Exposure to potentially restorative coastal blue–green places was unevenly distributed across Shenzhen. Residential areas with high or relatively high green-space exposure accounted for 16.12% of total residential area, whereas 167.31 km^2^, or 12.77%, was classified as low exposure. Coastal blue-space exposure was more concentrated: 95.71% of residential land fell within the lowest exposure category, while high and relatively high exposure accounted for only 0.49 and 0.70%, respectively. The lowest-exposure 80% of capacity-weighted residential locations accounted for 11.08% of total green-space exposure, and approximately 94.76% of capacity-weighted residential locations had almost no coastal blue-space exposure. High green-space exposure became rare at high social vulnerability levels, and no substantial blue-space exposure was observed among patches with SVI values above approximately 0.85.

**Conclusion:**

Socially vulnerable communities endure a compounded socio-environmental disadvantage, wherein systemic socioeconomic marginalization is exacerbated by severe spatial deprivation of everyday restorative blue–green exposures. This spatial mismatch underscores that the mere physical presence of urban nature does not equate to equitable health-promoting opportunities. To advance environmental health equity, urban governance must transcend macro-level ecological expansion to prioritize targeted, neighborhood-scale interventions. By integrating micro-spatial renewal, enhancing inclusive waterfront connectivity, and implementing robust anti-displacement safeguards, healthy-city planning can ensure that the salutogenic benefits of the urban exposome are equitably accessible to all residents.

## 1 Introduction

Urban blue–green spaces, defined as public green spaces such as urban parks and other related public greenery, as well as accessible coastal blue spaces, including shorelines and beaches, are increasingly regarded as everyday environmental exposures with implications for population health rather than as mere recreational amenities (1, 2). These environments are not only spatial resources but also places encountered, interpreted, and used through everyday routines (3). Consequently, these settings influence how residents move, cool down, interact socially, and obtain relief from urban stressors (4–8). Foundational research in environmental psychology and public health has consistently demonstrated that natural settings may be associated with directed-attention recovery, affective improvement, or reduced stress responses (9–12). Because spatial access serves as a precondition for realizing the positive impacts of blue–green spaces on residents, it is crucial to investigate whether residents’‌ geospatial locations facilitate access to these public amenities.

While the restorative potential of natural environments is well documented, these benefits materialize only when residents can practically access these spaces. Environmental justice research consistently demonstrates that urban nature is distributed unevenly across spatial and socioeconomic lines (13–15). Marginalized communities frequently experience reduced access to high-quality green spaces, raising concerns about distributional equity and green gentrification (16–19). Despite shorelines offering immense ecological and recreational value, coastal blue spaces have received less sustained attention (20–22). Although coastal environments are framed as shared urban assets, many residents rarely encounter the sea in their daily routines. This inequity is particularly pressing for socially vulnerable populations living in dense, lower-cost neighborhoods where environmental stressors are already concentrated (23–25). When these groups lack access to restorative blue–green environments, a severe double disadvantage emerges as heightened exposure to urban stressors is coupled with diminished place-based resources for recovery.

Shenzhen provides a revealing context in which to examine this problem (26–28). As one of China’s most rapidly urbanized coastal megacities, it combines intensive land development, a sharply differentiated housing market, and an extensive yet spatially uneven system of parks and coastal public spaces (29). High-value residential enclaves coexist with old urban villages, industrial districts, and newly developed peripheral communities (30–32). These contrasts are socioeconomic, but they are also experiential. They shape how far residents must walk, which public spaces can be entered with ease, and whether restorative environments become part of ordinary routines or remain occasional destinations. Fine-grained individual socioeconomic data are often difficult to obtain in Chinese megacities. At the residential-patch scale, however, housing prices, property-management fees, dwelling age, and surrounding commercial service structures can serve as useful spatial signals of social vulnerability, reflecting neighborhood status and the quality of everyday residential environments (33–37). These indicators reflect neighborhood status, service provision, market valuation, and the quality of everyday residential environments, while remaining closely connected to the home-based starting points from which daily exposure to restorative places usually unfolds.

Previous studies have examined green-space accessibility, blue-space accessibility, or neighborhood vulnerability separately, while many have relied on relatively coarse administrative units and Euclidean distance measures (38–40). This study extends that literature by linking place-based vulnerability, potential accessibility, and distributional inequality at the residential-patch level. Positioned at the intersection of environmental health and spatial justice, this study advances traditional frameworks through specific conceptual and methodological applications. Conceptually, rather than treating coastal blue–green spaces merely as physical amenities, the analysis examines them as essential components of residential environmental health. Methodologically, spatial models are deployed at a highly detailed residential scale. By utilizing diverse big data to construct a place-based contextual Social Vulnerability Index, this framework accurately identifies localized disparities in potential accessibility (41–43). It investigates whether residential areas with higher contextual vulnerability have more limited potential access to these health-promoting environments.

This study contributes to the literature by integrating green space and coastal blue space into a unified framework of place-based well-being opportunities, by proposing a residential-patch approach for identifying social vulnerability in a high-density city. Three questions guide this exploration: (1) how potential access to blue–green spaces is distributed, (2) whether vulnerable neighborhoods face limited access, (3) where socio-environmental deprivation emerges. Through this diagnostic framework, attention shifts from the total supply of urban nature to its everyday availability for different social groups, generating evidence for healthy city planning, neighborhood renewal, and more inclusive coastal public-space governance.

## 2 Research framework

### 2.1 Analytical framework

The study conceptualizes coastal blue–green environments as everyday components of the urban external exposome (44). The analytical framework links environmental health equity with residential exposure assessment by evaluating whether socially differentiated residential groups are equally positioned to encounter these settings. The framework executes four linked tasks: (1) identifying social vulnerability at the residential-patch scale, (2) estimating green-space exposure, (3) estimating coastal blue-space exposure, (4) and diagnosing place-based socio-environmental deprivation (Figure 1).

**Figure 1:**
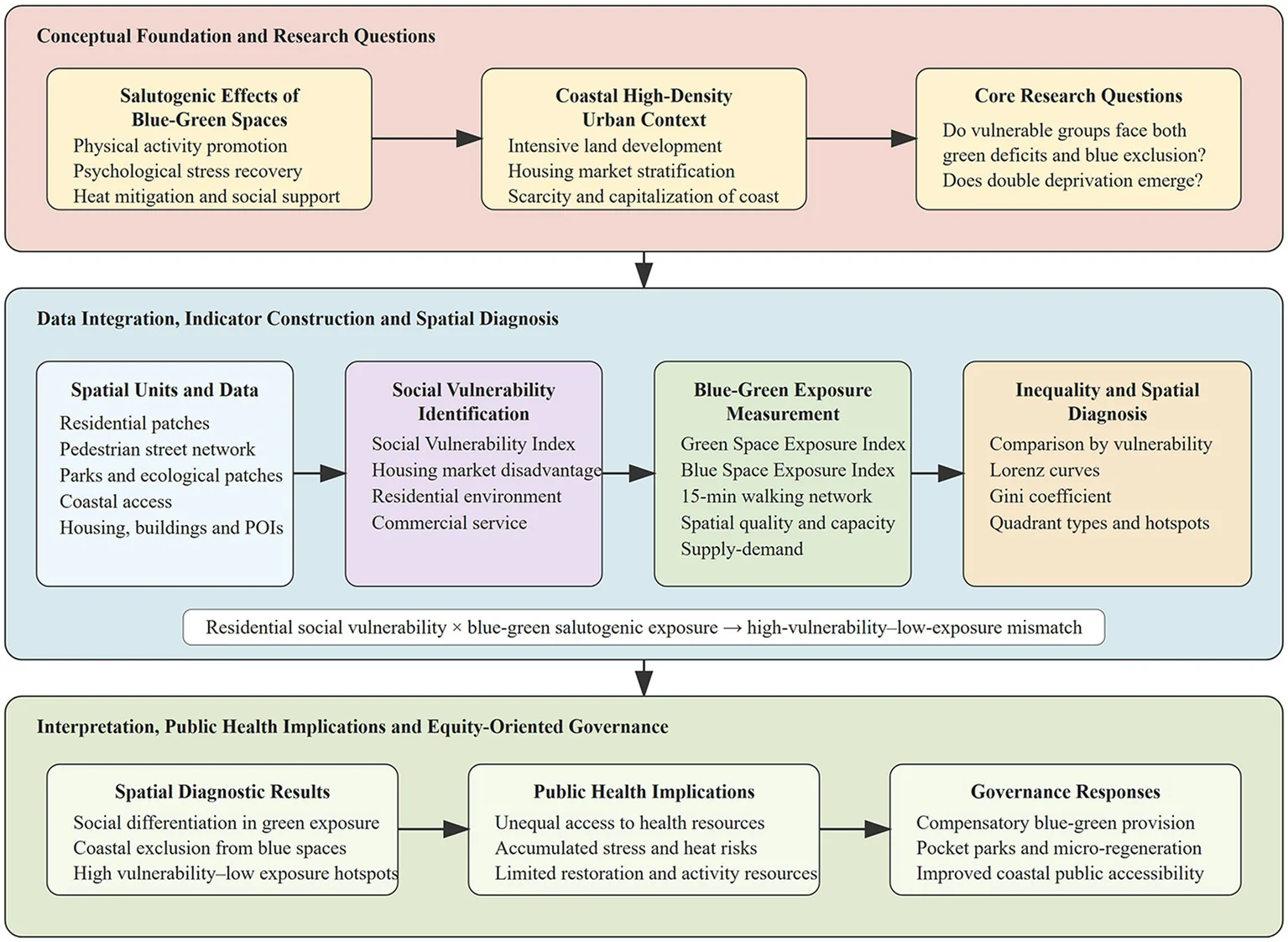
Analytical framework for assessing equitable exposure to blue–green salutogenic environments among vulnerable populations.

Social vulnerability is assessed at the residential-patch scale using indicators that capture housing market position, property-management conditions, dwelling age, and surrounding commercial-service structure. Exposure to green and coastal blue spaces is then evaluated within walking-network-based daily living circles. Rather than reducing access to straight-line distance from the nearest park or shoreline, the framework identifies whether blue–green places fall within a practical 15-min walking range from residential patches (45, 46).

By integrating these tasks, the study identifies neighborhoods where high social vulnerability overlaps with low blue–green exposure, aiming to inform health-oriented spatial governance by highlighting priority areas for targeted interventions.

### 2.2 Conceptual definition of potentially restorative blue–green places

Potentially restorative blue–green places are defined as publicly accessible green and coastal blue settings that contain environmental features commonly associated with psychological restoration, including vegetation, water, openness, visual relief, walkability, and partial separation from routine urban stressors (5, 6, 10, 12, 47). These ‌potential places are not assumed to be restorative for every user, and perceived restorativeness is not directly measured. This study operationalizes them as spatial settings that may afford emotion regulation and low-intensity social interaction when they are accessible in daily life (48).

### 2.3 Delineation of residential patches

Residential patches were used as the basic spatial unit because daily exposure to restorative environments usually begins from home. Residential land constitutes a fundamental component of urban construction, encompassing the spatial footprint of housing and synchronized service amenities. High precision identification of these specific parcels has been successfully achieved in existing literature, rendering them directly applicable for subsequent land use and spatial planning research (49). Polygons classified as residential land or residential compounds were extracted, while non-residential parcels, industrial land, commercial-only parcels, schools, hospitals, and public facilities were excluded. Adjacent residential polygons were dissolved when they formed the same compound or a continuous residential block. Polygons divided by arterial roads, rivers, railways, or fenced non-residential land were retained as separate patches, since these barriers may shape walking access and daily spatial experience. This process was completed using the GIS Dissolve tool restricted to singlepart features, which merged only polygons sharing physical boundaries and naturally preserved independent patches separated by barriers including roads and rivers. The final dataset contained 4,071 residential patches, providing a fine-grained basis for linking social vulnerability with blue–green exposure across Shenzhen.

## 3 Methods

### 3.1 Study area

Shenzhen is located in southern Guangdong Province, China (Figure 2), and has developed into a high-density coastal metropolis marked by rapid urban expansion, strong population concentration, and a highly differentiated housing market (26, 29). Decades of unprecedented demographic growth have generated severe spatial stratification, where affluent residential compounds seamlessly intermingle with densely populated low income urban villages. The city contains an extensive but uneven system of coastlines, coastal parks, urban parks, and greenways (16, 27, 28). This profound social heterogeneity overlapping with abundant natural amenities establishes the region as an ideal laboratory to investigate environmental justice. The extreme residential polarization ensures that diverse vulnerability profiles.

**Figure 2:**
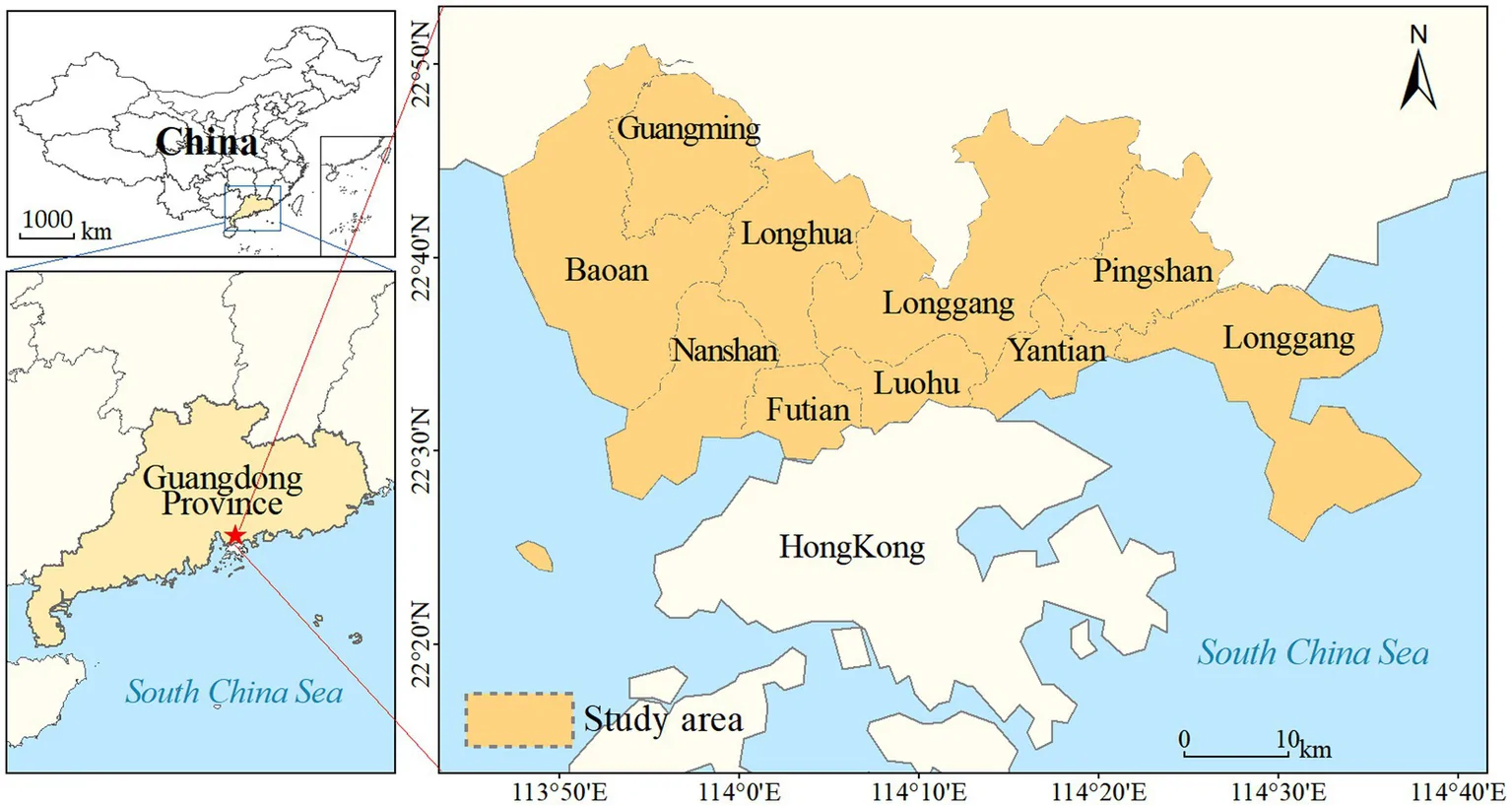
Geographical location of the study area.

The city also offers relatively rich multi-source spatial data, including land-use classification, road networks, urban parks and green spaces, coastal public spaces, building footprints, points of interest, and road distribution data (Table 1). The empirical records gathered across the study area exhibit absolute comprehensiveness, ensuring our analytical framework encountered absolutely no missing values. These datasets were used to characterize vulnerable residential areas, blue–green space provision, walking accessibility, and potential service pressure at a fine spatial scale. They also made it possible to identify residential-scale disparities in exposure to potentially restorative environments and to examine where environmental deprivation may overlap with social vulnerability.

**Table 1:** Data sources and descriptions.

Data type	Data content	Data source	Year	Spatial scale	Main use
Administrative boundary data	Shenzhen city, review number GS(2024)0650	Ministry of Natural Resources of China	2024	Vector polygon	Study area boundary
Green space data	Urban parks, grasslands and other green space patches	Published literature (49)	2025	Vector polygon	Measurement of green-space exposure
Blue space data	Coastlines	Published literature (49)	2025	Vector polygon	Measurement of blue-space exposure
Road network data	Urban roads, pedestrian paths and street networks	OpenStreetMap, extracted via OSMnx/Overpass API (https://www.openstreetmap.org)	2024	Vector line	Network-based accessibility analysis
Housing market situation	Residential housing prices, property management price, and age of the house	Anjuke real estate platform (https://www.anjuke.com/)	2025	Vector point	Measuring housing market conditions, residential cost, and built-environment aging
Building height	The height of the building, measured, meters	UT-GLOBUS dataset, Zenodo (78)	2024	Vector polygon	Characterize residential capacity
Commercial service facilities	Cafés, yoga studios, import specialty stores, budget hotels, convenience stores, lottery retailers et al.	Amap Open Platform (https://lbs.amap.com/)	2025	Vector point	Characterize the consumption level of residential areas

### 3.2 Quantitative evaluation of the social vulnerability index

#### 3.2.1 Construction of the social vulnerability index

This study adapts the proxy-based spatial Social Vulnerability Index to the residential-patch level to address the limited spatial resolution of census-based and survey-based indicators. In high-density cities, aggregated data may conceal substantial variation among residential areas, while person-level socioeconomic data are rarely available at this scale. We therefore use locally available indicators, including housing prices, dwelling age, property-management fees, and commercial density, to estimate relative, place-based socioeconomic vulnerability. The index characterizes residential contexts rather than individual residents, enabling a finer assessment of living environments and localized disparities in access to blue–green spaces.

Fine-scale socioeconomic data are not publicly available for individual residential compounds in Shenzhen. An area-level social vulnerability index (SVI) was therefore constructed using housing-market, property-management, dwelling-age, and neighborhood-service proxies (Table 2). Comprehensive descriptive statistics encompassing the mean, standard deviation, minimum, and maximum values for all raw variables prior to normalization have been incorporated into Table 2 to ensure complete data transparency. All indicators were standardized to a range of [0, 1] through Min–Max normalization. Dwelling age and commercial service structure were transformed utilizing the Equation 1, while housing market metrics and property management fees required the application of Equation 2. The baseline SVI was calculated with equal weights, since no established empirical evidence indicates that one proxy should dominate the others in the Shenzhen context. The equal weighting approach was adopted to preserve the theoretical equivalence of the four selected indicators, preventing the numerical distortions typical of variance-driven statistical techniques and the subjective biases inherent in expert-scoring methods (50–53).

$$N^{\prime }=\frac{N-N_{\min}}{N_{\max}-N_{\min}}$$ (1)

$$N^{\prime }=\frac{N_{\max}-N}{N_{\max}-N_{\min}}$$ (2)

wherein *N′* and *N* are normalized and calculated values, respectively; *N_max_* and *N_min_* represent maximum and minimum of calculation values of the index in all individual residential compounds, respectively.

**Table 2:** Indicators used to construct the social vulnerability index.

Dimension	Variable	Direction in SVI	Rationale	Mean	Standard deviation	Minimum	Maximum
Housing market	Mean housing price (CNY/m^2^)	Lower price = higher vulnerability	Proxy for purchasing power and neighborhood market value	57984.030	23463.737	8,351	235,429
Property management	Monthly property fee (CNY/m^2^/month)	Lower fee = higher vulnerability	Proxy for formal maintenance intensity	2.325	1.266	0.963	37.080
Dwelling age	Mean housing age (Years)	Older = higher vulnerability	Proxy for physical ageing and outdated facilities	20.876	4.581	4	71
Commercial-service structure	Low-end service ratio or high-end service scarcity (POI-based index)	Higher low-end ratio = higher vulnerability	Proxy for neighborhood consumption landscape	0.302	0.275	0	1

The selected proxies capture different dimensions of relative residential vulnerability. In the highly stratified context of Chinese megacities, these physical and economic metrics extend well beyond market values to fundamentally shape spatial inequality. Housing price reflects accumulated market valuation and household purchasing power (52). Lower property values concentrate low income families whose inadequate savings and social provisions severely diminish their economic buffer against shocks. Property-management fee indicates the intensity of formal maintenance and estate-management quality. Insufficient management fees dictate severe maintenance deficits, which degrade essential safety infrastructure and leave emergency response capabilities considerably compromised by restricted service supplies. Dwelling age captures potential physical deterioration and older neighborhood infrastructure (23, 36). Aging communities couple degraded infrastructure with concentrated marginalized populations burdened by heightened environmental sensitivity and significantly weakened recovery capabilities. Local commercial-service structure reflects the consumption landscape and neighborhood socioeconomic status. After eliminating spatial duplicates, raw commercial locations were filtered categorizing cafés, yoga studios and import specialty stores as high-end amenities while designating budget hotels, convenience stores and lottery retailers as low-end facilities to calculate the precise proportion of low-end point positions. When surrounding facilities are restricted entirely to basic living necessities, they reveal depressed community consumption power and sluggish economic vitality. Recent urban computing studies validate commercial POIs as robust socioeconomic proxies in Chinese cities (54). These variables do not measure individual poverty directly. When superimposed, however, these overlapping disadvantages leave residents highly susceptible to external disruptions. Their compromised ability to cope and recover fundamentally drives up the overall social vulnerability across these residential environments. Extensive literature in urban geography and public health consistently validates that housing prices serve as robust proxies for micro-level socioeconomic and neighborhood characteristics, particularly when fine-grained census data are unavailable (33, 37, 55, 56).

#### 3.2.2 Assessment of indicator redundancy

Prior to aggregating the four constituent indicators into the social vulnerability index, potential redundancy among the indicators required rigorous evaluation across all residential patches. All analytical variables were directionally aligned ensuring higher values consistently reflected greater vulnerability.

Pairwise Spearman rank correlations initially evaluated direct bivariate relationships. Values of ∣ρ∣ ≥0.80 were treated as evidence of potentially substantial pairwise redundancy. Auxiliary regressions projecting individual indicators against the remaining three provided a complementary multicollinearity diagnostic calculating the variance inflation factor (VIF). VIF was calculated as VIF*_j_* = 1/(1 − *R_j_*^2^), where *R_j_*^2^ denotes the coefficient of determination from the auxiliary regression. Tolerance was calculated as 1 − *R_j_*^2^. Values of VIF above 5 were considered indicative of potentially problematic multicollinearity (57). The maximum observed Spearman correlation occurred between housing price and commercial service structure (*ρ* = 0.333) (Supplementary Table S1). Diagnostic VIF outputs ranged from 1.005 to 1.119 confirming no parameter breached the critical limits (Supplementary Table S2). Empirical results demonstrated minimal redundancy.

Previous studies have shown that urban villages tend to contain disproportionately large shares of low-income, migrant, and otherwise socially disadvantaged populations (58–61). Importantly, the urban-village classification was not used to construct, weight, or calibrate the SVI. We therefore tested whether the SVI assigns higher scores to urban villages than to formal neighbourhoods. A Mann–Whitney U test revealed that SVI scores were significantly higher in urban villages than in formal neighborhoods (U = 824,435, Z = −18.050, *p* < 0.001). Additionally, ROC curve analysis showed acceptable discriminative ability in identifying these vulnerable areas, yielding an Area Under the Curve of 0.721 (*p* < 0.001). These results provide moderate empirical support for the known-groups validity of the SVI.

### 3.3 Measuring exposure to potentially restorative blue–green places

An enhanced two-step floating catchment area model was used to estimate exposure to potentially restorative blue–green places (40, 62, 63). The model was calculated separately for public green spaces and public coastal blue spaces. The exposure index was specified as a modified supply–demand measure that integrates walking-network accessibility, environmental service capacity, and potential residential demand (64). The blue–green space exposure index is calculated using Equations 3–6 as follows:

$$R_{j}^{k}=\frac{A_{j}^{k}}{\sum_{i\epsilon C_{j}}P_{i}B_{\mathrm{ij}}}$$ (3)

$$E_{i}^{k}=\sum_{j\epsilon C_{i}}R_{j}^{k}B_{\mathrm{ij}}$$ (4)

$$B_{\mathrm{ij}}=\{\begin{array}{c} 1,T_{\mathrm{ij}}\leq T_{0}\\ 0,T_{\mathrm{ij}}>T_{0} \end{array}$$ (5)

$$P_{i}=BF_{i}\times BH_{i}$$ (6)

where *k* denotes the type of blue–green space, $R_{j}^{k}$ represents the supply–demand ratio of blue–green space *j*, *B_ij_* is the binary walking-accessibility indicator between residential patch *i* and blue–green space *j*; $E_{i}^{k}$ denotes the blue–green space exposure index of residential patch *i*, $A_{j}^{k}$ represents the area of blue or green space, *P_i_* represents the residential capacity of patch *i*, *C_j_* denotes the service catchment of blue–green space *j*, it represents the 15-min living circle threshold. *T_ij_* is the walking time between residential patch *i* and space *j*, *T_0_* is the 15-min threshold. *BF_i_* and *BH_i_* represent the building footprint area and the average building height of the *i*-th residential patch, respectively. The calculation of *Pi* was introduced to determine the total residential building area at the patch scale, which allows us to approximate the potential population demand for blue–green spaces across different patches. Recognizing metropolitan tolerance for recreational crowding the model assumes baseline park capacities adequately fulfill local demands without incorporating complex overcrowding penalties.

All spatial operations and network distance calculations were executed entirely within the native geoprocessing environment of ArcGIS Pro 3.7 utilizing the WGS 1984 UTM Zone 50 N coordinate reference system. The spatial analysis defined intersection points between residential patches and the street network as origin nodes while designating the corresponding road connections of green spaces or coastlines as destination nodes, thereby excluding inaccessible private waterfronts. Origin–destination cost matrices were subsequently generated through the network analysis tool to compute precise walking distances along the pedestrian road network. Assuming an average adult walking speed of 1.2 meters per second established a maximum walking network distance threshold of 1,080 meters (65, 66). Topographical variations were assumed to exert no substantial influence on pedestrian accessibility considering the spatial analysis restricted the maximum travel threshold to a relatively short distance of 1,080 meters. This specific spatial limit accurately delineates the 15-min living circle designated to evaluate daily environmental exposure.

### 3.4 Inequality and place-based deprivation analysis

Inequality in blue–green exposure was examined through descriptive comparison, distributional analysis, and spatial diagnosis (17, 20, 62). To facilitate this, the continuous green-space and blue-space exposure indices were classified into five levels using the geometric interval method: low, relatively low, medium, relatively high, and high. This specific algorithmic approach optimally accommodates the highly skewed distribution, ensuring visual contrast while maintaining statistical validity across unevenly dispersed data ranges. These categorized exposure levels were then compared across residential patches with different levels of social vulnerability, allowing broad exposure gradients to be identified before spatial clustering was assessed.

Lorenz curves were then used to evaluate the overall distribution of exposure. Residential patches were sorted from the lowest to the highest exposure score. The horizontal axis represents the cumulative share of residential capacity, while the vertical axis represents the cumulative share of total exposure, calculated as *P_i_* × *E_i_*. The resulting inequality estimates therefore describe exposure distribution across capacity-weighted residential locations rather than individual survey respondents. The Lorenz curves quantify the imbalance between cumulative residential capacity and cumulative exposure to potentially restorative blue–green environments. To provide a precise mathematical measure of this imbalance, the *Gini* coefficient was calculated based on the Lorenz curves (67). The value of the *Gini* coefficient ranges from 0 to 1, where 0 indicates perfect equality and 1 represents absolute inequality.

Scatterplots with marginal distributions were used to explore the relationship between social vulnerability and blue–green exposure at the neighborhood scale. This visual approach helped identify nonlinear patterns that may be obscured by mean comparisons. Quadrant typology analysis and bivariate spatial mapping were further applied to identify residential patches where high vulnerability coincides with low blue–green exposure. For the quadrant classification, thresholds were established based on the median values of the social vulnerability index and the respective exposure metrics. This divided residential patches into four socio-environmental types: high vulnerability–low exposure, low vulnerability–high exposure, low vulnerability–low exposure, and high vulnerability–high exposure. Patches falling into the high vulnerability–low exposure quadrant were defined as place-based deprivation zones (24, 68). Such areas were treated as priority locations for equity-oriented planning, neighborhood renewal, and coastal public-space intervention.

### 3.5 Sensitivity analysis

To evaluate the robustness of the spatial accessibility outcomes, a sensitivity analysis was incorporated into the methodology. The E2SFCA model was executed across four varying pedestrian catchment scenarios representing 10-min, 15-min, 20-min, and 30-min walking thresholds. Analytical focus was directed toward comparing the resulting Lorenz curves alongside the identification of deprivation hotspots characterized by high social vulnerability and low environmental exposure (Supplementary Figure S1). While larger catchment areas naturally produced higher absolute exposure values, the relative inequality metrics exhibited remarkable stability. The consistent spatial distribution of disadvantaged communities across all four scenarios suggests that the main findings are not highly sensitive to the selected walking-threshold specification. Following this methodological validation, the 15-min walking threshold was definitively established as the primary baseline for the final evaluation. This specific temporal limit perfectly matches mainstream practices in contemporary environmental studies while firmly adhering to the 15-min community life circle guidelines comprehensively institutionalized across Chinese urban planning disciplines (45, 69–71).

### 3.6 Statistical and spatial autocorrelation analyses

Inferential statistics and spatial autocorrelation models were introduced to rigorously validate socioenvironmental disparities. Spearman correlation was selected because the underlying spatial datasets display non normal distributions. Residential patches lacking blue space access were completely excluded to address zero inflation phenomena associated with localized water bodies. A stratified analytical framework categorized the city into core urban, near suburban, and far suburban zones which enables a nuanced evaluation of green space exposure across varying developmental contexts. The global Moran index was subsequently applied to determine whether social vulnerability and environmental metrics exhibit significant spatial agglomeration (72). Positive index values accompanied by statistically significant *p* values confirm that disadvantaged neighborhoods form distinct geographical clusters establishing a robust mathematical foundation for localized hotspot identification.

## 4 Results

### 4.1 Spatial heterogeneity of exposure to coastal blue–green places

Based on 4,071 residential patches in Shenzhen, the results reveal marked spatial heterogeneity in residents’ exposure to potentially restorative blue–green places (Figure 3). Although the city contains a large system of parks, greenways, coastal landscapes, and waterfront public spaces, these resources are not evenly embedded in everyday residential environments. Physical distance, fragmented pedestrian routes, uneven service capacity, and spatial barriers jointly shape whether natural settings can become usable places for routine restoration.

**Figure 3:**
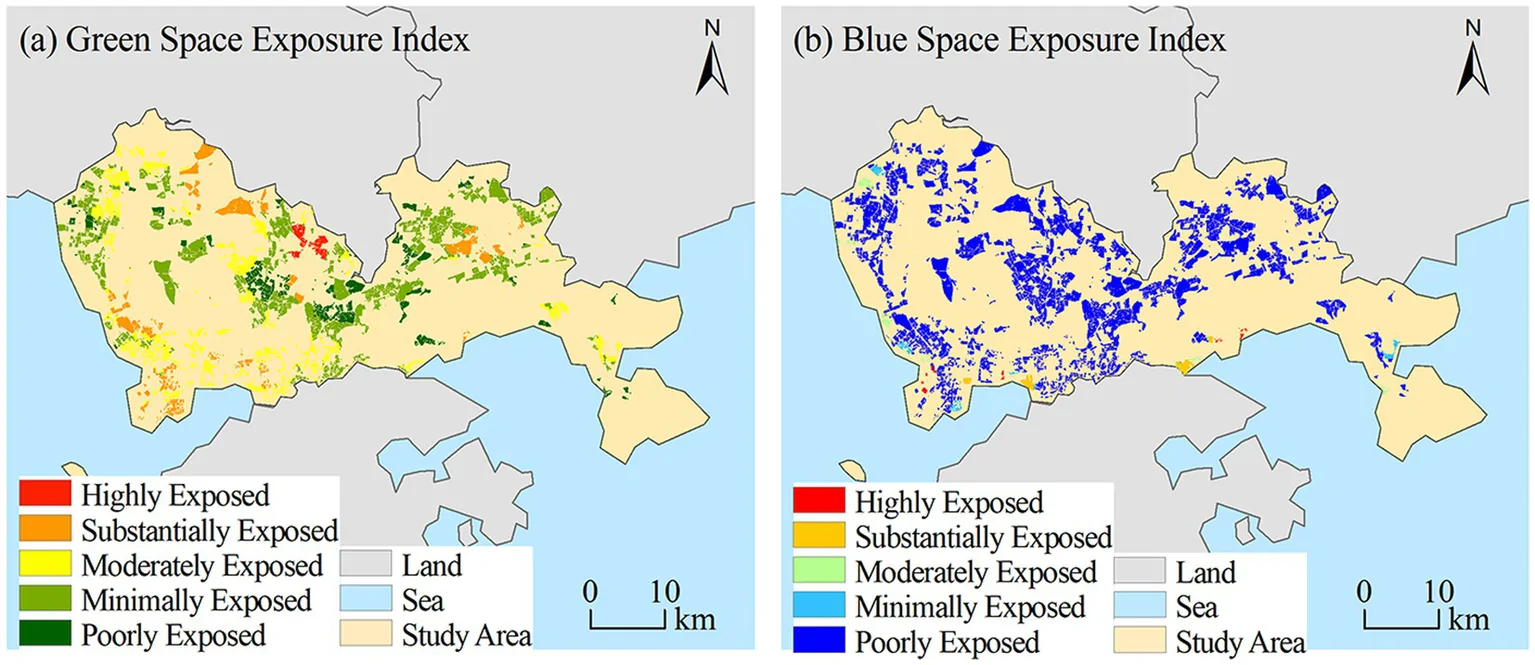
Spatial distribution of blue/green exposure levels in Shenzhen. The continuous **(a)** green-space and **(b)** blue-space exposure indices were classified by the geometric interval method.

Green-space exposure shows a clear but fragmented spatial gradient (Figure 3A). Residential areas with high and relatively high exposure account for only 16.12% of the total residential area. These patches are not continuously distributed; they appear as scattered clusters near large ecological cores, major parks, mountain-edge landscapes, or well-connected green corridors. By contrast, 167.31 km^2^ of residential land falls into the low-exposure category, representing 12.77% of the total residential area. Shenzhen’s greening efforts have improved the city’s overall ecological landscape, yet accessible and usable green environments remain unevenly connected to daily residential life. For many neighborhoods, nearby opportunities for walking, informal social contact, quiet rest, and psychological restoration are still limited.

Blue-space exposure displays a much sharper spatial contrast (Figure 3B). Although Shenzhen is a coastal metropolis, 95.71% of its residential land has very low exposure to publicly accessible coastal blue spaces. Areas with high and relatively high blue-space exposure are extremely limited, accounting for only 0.49 and 0.70% of total residential land, respectively. They are concentrated mainly along the southern coastline, while exposure declines rapidly as distance from the coast increases. The sea is therefore prominent in Shenzhen’s spatial identity, but its restorative affordances are largely confined to the first coastal belt. For most inland neighborhoods, coastal walking routes, waterfront views, sea breezes, and shoreline settings are unlikely to form part of ordinary daily living circles.

### 4.2 Inequality across the social gradient: quantifying environmental health deficits

#### 4.2.1 Spatial concentration of blue–green restorative affordances

The empirical Lorenz curves deviate substantially from this benchmark, indicating that place-based well-being opportunities are highly concentrated (Figure 4). The degree of concentration differs between green and blue spaces, with coastal blue exposure showing the more extreme inequality. The calculated *Gini* coefficient for green-space exposure was 0.858, whereas coastal blue-space exposure exhibited a near-absolute inequality with a *Gini* coefficient of 0.992.

**Figure 4:**
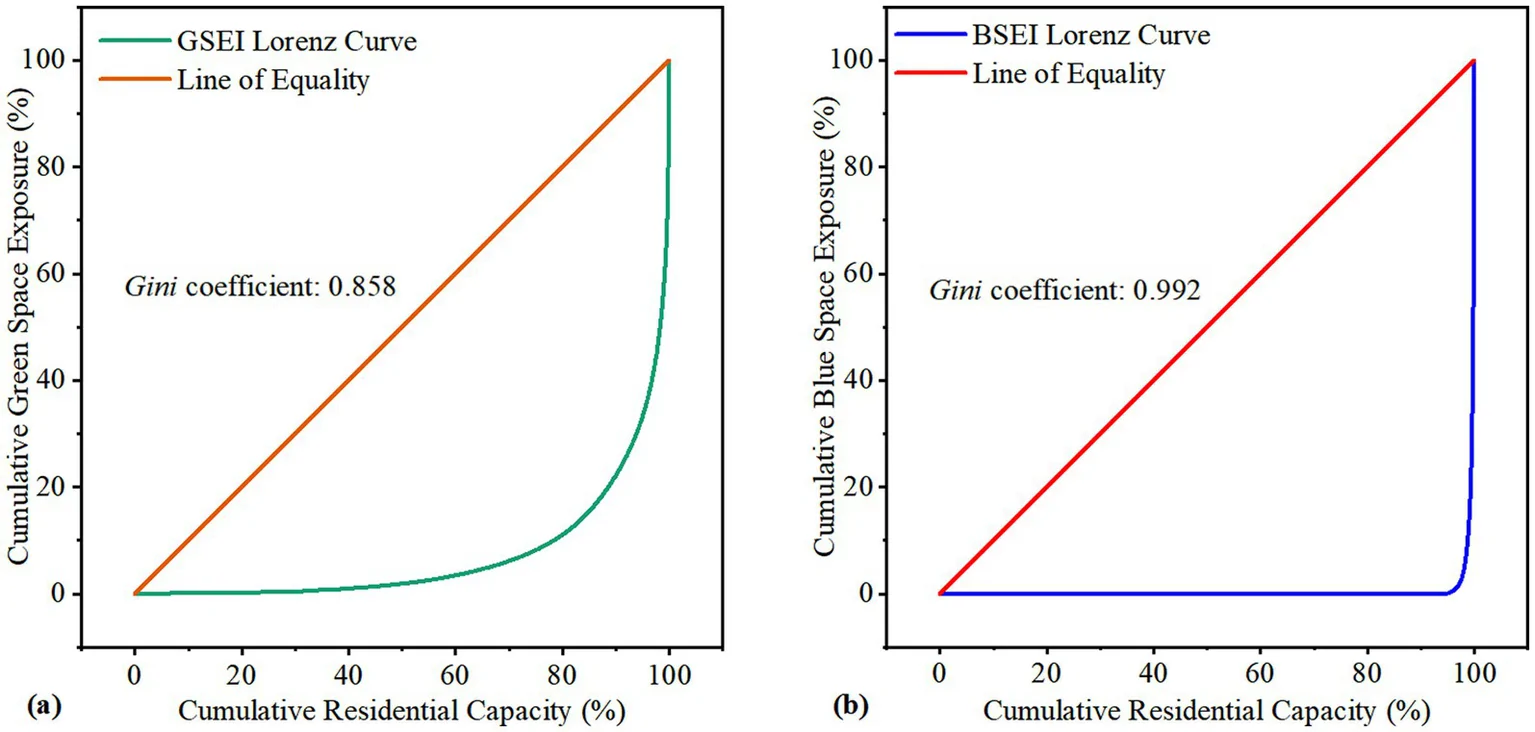
Lorenz curves of exposure opportunities to blue–green restorative environments. **(a)** Lorenz curve of green-space exposure. **(b)** Lorenz curve of blue-space exposure. Under perfect equality, the curve would overlap with the diagonal line. GSEI, Green Space Exposure Index, BSEI, Blue Space Exposure Index.

Green-space exposure presents a strong concentration pattern. As shown in Figure 4A, the Lorenz curve is markedly concave in the lower and middle sections, meaning that most capacity-weighted residents share only a small proportion of total green-space exposure. After residential patches were sorted by exposure score, the lowest-exposure 80% of capacity-weighted residents accounted for only 11.08% of total green-space exposure. High-quality green exposure is therefore concentrated in a limited number of residential areas rather than being broadly available across the city’s daily living environments. Parks and green spaces may exist within the urban system, but for many communities they remain weakly connected to routine use.

Inequality in coastal blue-space exposure is even more pronounced. The Lorenz curve has an L-shaped form and remains close to the x-axis until the cumulative residential capacity reaches 94.76% (Figure 4B). In practical terms, about 95% of the capacity-weighted population has almost no daily exposure to publicly accessible coastal blue spaces. Most blue-space exposure opportunities are concentrated among the top 5%.

#### 4.2.2 Nonlinear relationships between social vulnerability and blue–green exposure

While the Lorenz curves reveal citywide distributional inequality, the scatterplots between the social vulnerability index and blue–green exposure indices show how this inequality appears at the neighborhood scale (Figure 5). The social vulnerability index is approximately skewed normally distributed, spanning from 0.14 to 0.94 with a mean value of 0.62 and a standard deviation of 0.12, ultimately representing a continuous gradient of relative socioeconomic disadvantage across residential environments, whereas green space exposure exhibits a clear zero inflated pattern within the marginal histogram (Figure 5A). Many communities cluster at the lowest exposure levels. The relationship between vulnerability and green-space exposure is not a simple linear decline; it forms a wedge-shaped distribution with a visible ceiling effect.

**Figure 5:**
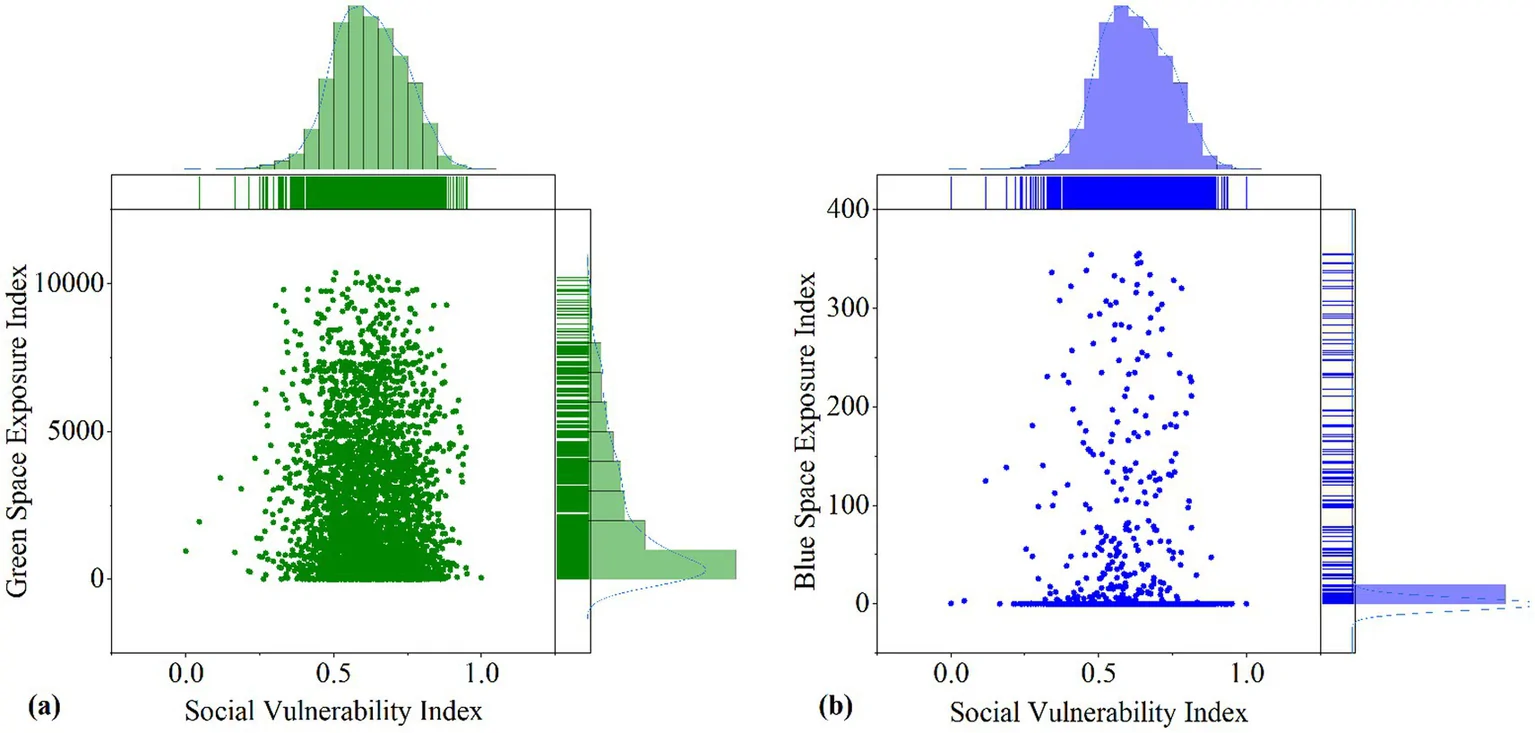
Nonlinear relationships between social vulnerability and blue–green space exposure. **(a)** Relationship between the social vulnerability index and green-space exposure index. **(b)** Relationship between the social vulnerability index and blue-space exposure index.

In residential areas with low and medium vulnerability, green-space exposure varies widely, ranging from zero to more than 10,000. Less vulnerable groups therefore appear to occupy more diverse residential environments: some benefit from high-quality green exposure, while others do not. This variability narrows sharply at the high end of vulnerability. When the vulnerability index rises above approximately 0.7 and approaches 1.0, high green-space exposure values almost disappear, and residential patches become concentrated at low exposure levels. Severe green-space deprivation is thus most likely to occur among highly vulnerable communities. Older, lower-cost, or less well-managed neighborhoods tend to have more constrained access to green places that support daily movement, informal interaction, and quiet restoration. Inferential statistical tests confirm these descriptive spatial patterns by revealing distinct inequalities in resource distribution. A stratified analysis of green space yields even more pronounced disparities within specific developmental contexts. The core urban area demonstrates a highly significant negative correlation (*p* < 0.01, *r* = −0.461). Such a strong statistical relationship mathematically confirms that environmental gentrification and resource deprivation are particularly acute in the most densely developed sectors of the city.

The scatterplot for blue-space exposure shows an even more compressed pattern (Figure 5B). Because accessible coastal blue resources are spatially limited, nearly 95% of residential areas have exposure values close to zero regardless of vulnerability level. Only a small number of communities show very high blue-space exposure, and most of them fall within the medium-to-low vulnerability range, with SVI values between approximately 0.4 and 0.7. Among the most vulnerable residential areas, where the SVI exceeds 0.85, blue-space exposure is absent. To rigorously test this observation patches with accessible water bodies were isolated for further evaluation. These locations display a significant negative correlation between social vulnerability and blue-space exposure (*p* < 0.05, *r* = −0.210). Highly vulnerable populations experience substantially lower quality or quantity of blue-space access even when such resources are present in their general vicinity.

### 4.3 Spatial mismatch and identification of place-based deprivation hotspots

The preceding analyses show that unequal exposure to restorative blue–green environments is not only a citywide distributional issue; it also has a distinct neighborhood geography. Global spatial autocorrelation analysis perfectly validates the clustered nature of these socioenvironmental disparities before delving into specific neighborhood mismatches. The Global Moran’s I indices calculated for social vulnerability, green space exposure, and blue space exposure are 0.219, 0.345, and 0.227, respectively. Inferential statistical evaluation reveals that all three variables display highly significant positive spatial clustering with Z values reaching 56.756, 90.812, and 60.292 while *p* values remain consistently less than 0.001. Vulnerable communities and restorative environmental resources are clearly not scattered randomly across the metropolitan landscape. They instead form concentrated spatial agglomerations where specific conditions tend to geographically aggregate. Such macro level statistical confirmation provides rigorous justification for the subsequent bivariate hotspot classification which pinpoints precise residential patches facing overlapping disadvantages. The four socio-environmental types represent a different combination of residential disadvantage and restorative environmental opportunity (Figure 6).

**Figure 6:**
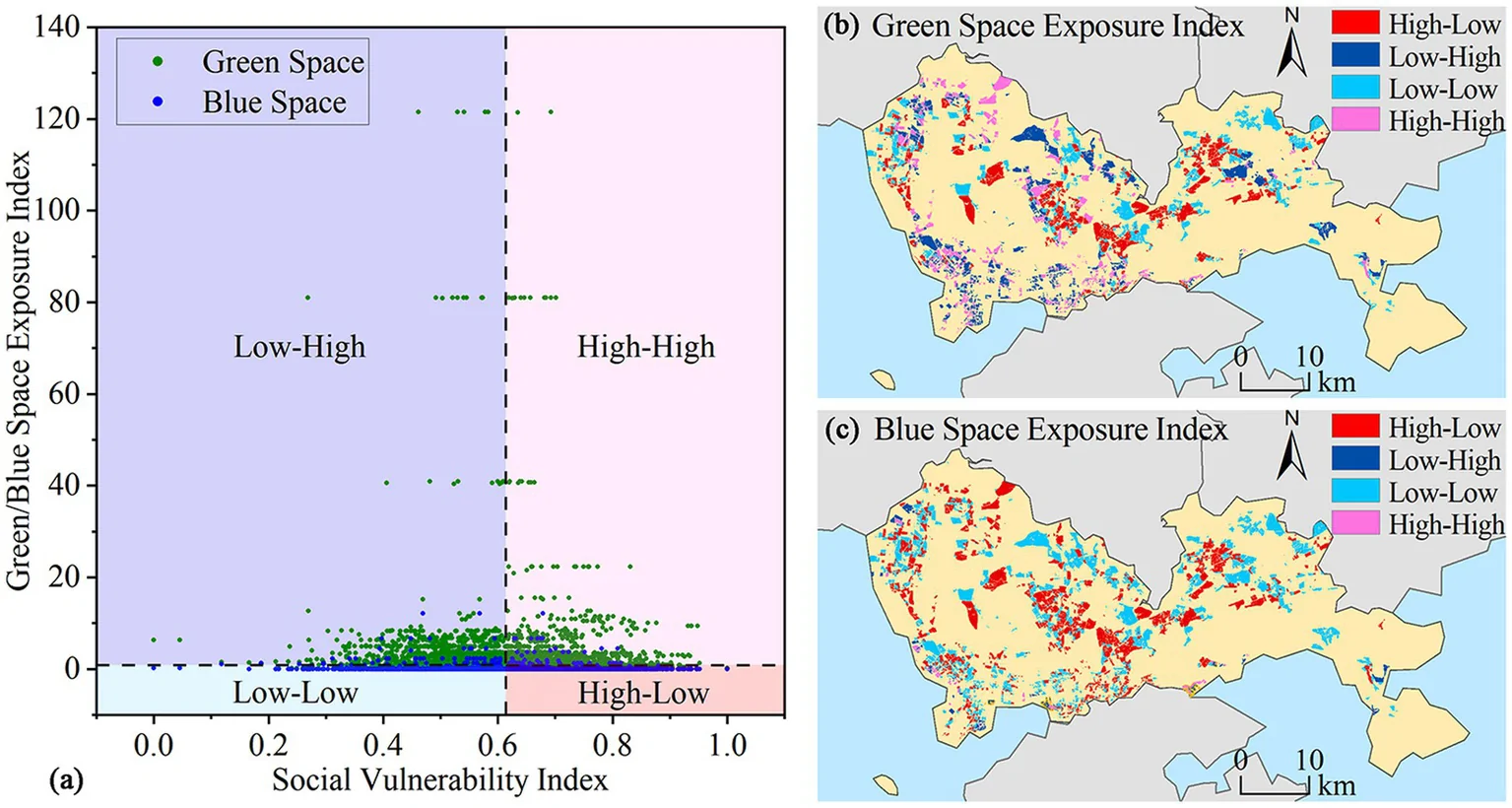
Bivariate spatial typology of social vulnerability and blue–green space exposure. **(a)** Quadrant classification of social vulnerability and blue–green space exposure indices. **(b)** Spatial distribution of green-space exposure typology. **(C)** Spatial distribution of blue-space exposure typology. The thresholds were established based on the median values of the social vulnerability index and the respective exposure metrics.

The High–Low category is the central concern of this study. It captures areas where high social vulnerability coincides with low exposure to blue–green restorative places. For green-space exposure, many observations fall within the high-vulnerability quadrant (Figure 6A). In total, 49.70% of high-vulnerability communities are classified as High–Low, indicating that nearly half of socially vulnerable communities experience very limited green-space exposure. Spatially, these red “double-burden”‌ hotspots are not randomly distributed (Figure 6B). Many are concentrated in old and dense central districts, while others appear in northern peripheral industrial areas. These neighborhoods often combine high building density, limited redevelopment space, lower property-management fees, and long-term underinvestment in small public spaces.

Regarding green spaces, the Low–High areas account for 24.02% of communities and are mainly located near large country parks and mountain landscapes. Meanwhile, 18.28% of communities fall into the High–High category (Figure 6B). For blue-space exposure, however, the Low–High type is far less common (Figure 6c). It appears only in scattered locations along the southern coast, consistent with the highly compressed distribution of blue-space exposure shown earlier. Blue-space privilege in Shenzhen is therefore spatially narrow rather than widespread.

Taken together, the bivariate results show that Shenzhen’s blue–green exposure inequality is shaped by both scarcity and mismatch. Green-space deprivation is concentrated in specific vulnerable neighborhoods, whereas blue-space deprivation is almost citywide and becomes most absolute among the most vulnerable communities. Restorative place opportunities are therefore unevenly embedded in Shenzhen’s residential landscape.

## 5 Discussion

### 5.1 From spatial presence to everyday blue–green exposure opportunities

This study extends environmental health and environmental equity research by shifting attention from the mere physical presence of blue–green spaces to their function as route-dependent and residentially anchored opportunities for everyday environmental contact. Consistent with international research showing that visual and water-related natural settings can receive favorable affective or potentially restorative evaluations, while responses vary according to environmental attributes and user experience (73–75), the present findings suggest that the public-health relevance of such environments depends not only on whether they exist within the city, but also on whether they can be reached through ordinary walking routines.

This perspective complements earlier assessments based on Euclidean distance, circular buffers, or aggregate area, because walking-network measures can identify barriers and route constraints that remain invisible in purely geometric representations (66). It also extends the literature on park-access inequalities, which has largely focused on green-space provision in Western urban contexts (15, 19, 67), by showing that in a hyper-dense Asian megacity the modeled opportunity for everyday coastal blue-space exposure may be more spatially constrained than that for green-space exposure. Green and coastal blue environments should therefore not be treated as interchangeable indicators of “nature”: the former were distributed across multiple ecological and urban settings, whereas the latter were concentrated within a relatively narrow shoreline belt and depended more strongly on shoreline configuration, route connectivity, destination capacity, and surrounding land use.

For residents in lower-access residential areas, these spatial patterns may correspond to fewer opportunities to encounter environments that previous studies have associated with physical activity, thermal comfort, or psychological restoration; however, because this study did not measure behavior, perceived restoration, or health outcomes, it does not establish that accessibility caused restoration or improved health. Rather, its contribution lies in identifying where potentially health-relevant blue–green exposure opportunities are more or less available within daily activity spaces, and in demonstrating why green and coastal blue environments should be examined as distinct components of the urban exposome rather than aggregated into a single measure of environmental provision.

### 5.2 Environmental justice and the relational nature of green-space access

The association between social vulnerability and green-space exposure is consistent with the broader environmental-justice literature. Studies in different national settings have reported socioeconomic or racialized inequalities in proximity to, availability of, or access to urban parks (13, 15). Mitchell and Popham (14) further showed why the distribution of natural environments matters in discussions of health inequality. The Shenzhen results add a residential-scale perspective to this literature by showing how vulnerability proxies and network-based environmental access can be examined within the same analytical unit.

The findings also support a relational understanding of environmental privilege. A publicly designated park or shoreline is not necessarily an equally available resource, since housing prices may capitalize on environmental amenities, while arterial roads, railways, gated developments, topography, limited entrances, and weak pedestrian connections can restrict practical accessibility. Maintenance quality and social conditions may further influence who feels able or welcome to use a place. This interpretation is consistent with the just green enough planning approach discussed by Wolch et al. (19) and with the argument presented by Jennings et al. (18) that environmental justice extends beyond the formal distribution of amenities. Spatial access, procedural inclusion, recognitional justice, and protection from displacement therefore need to be considered together.

The high-vulnerability/high-exposure category is also theoretically important. It indicates that social vulnerability does not always coincide with lower measured environmental exposure. Some lower-cost or older residential areas may be located close to historical parks or ecological corridors. These cases complicate a simple assumption that vulnerable communities are always located far from environmental resources. They also suggest that protecting existing accessible green spaces may be as important as constructing new ones.

### 5.3 Implications for exposome-informed healthy City planning

Addressing spatial disparities requires moving beyond traditional supply-led planning toward targeted interventions informed by the urban exposome. Generic recommendations to improve walkability prove insufficient in high-density contexts. Municipal planners should instead deploy specific spatial strategies within identified high-vulnerability and low-exposure hotspots. These specific patches serve as screening zones for detailed pedestrian audits rather than automatic large-scale redevelopment sites. The district-level distribution of these areas is summarized in Table 3. Reporting both absolute area and the proportion of district residential area helps distinguish districts with the largest total extent from those in which this spatial pattern is most concentrated. These statistics can support the local interventions.

**Table 3:** District-level distribution of residential areas with high vulnerability and low green-space exposure in Shenzhen.

District	Urban location category	High vulnerability and low exposure area/km^2^	Proportion within district residential area/%	Share of citywide high vulnerability and low exposure area/%	Number of spatial patches
Futian	Central urban area	5.633	40.450	8.530	189
Luohu	Central urban area	4.995	31.023	7.564	159
Nanshan	Central urban area	8.110	35.678	12.280	173
Baoan	Inner suburban area	17.401	20.737	26.348	236
Longhua	Inner suburban area	10.861	18.879	16.445	81
Longgang	Inner suburban area	4.942	4.073	7.484	32
Yantian	Inner suburban area	1.003	22.342	1.519	15
Guangming	Outer suburban area	11.974	37.123	18.131	71
Pingshan	Outer suburban area	1.123	3.761	1.700	14

Within these deprived neighborhoods, micro-scale urban acupuncture offers a highly practical solution. Local agencies can retrofit residual urban parcels into pocket parks and unlock fenced institutional greenery for carefully managed shared public use (42). Small corrective projects can significantly increase practical access without requiring extensive land acquisition. Such targeted improvements include installing continuous sidewalks, adding barrier-free ramps, providing adequate lighting, and subsidizing community-led maintenance to ensure existing spaces remain safe and usable throughout the day.

Coastal planning demands an entirely different approach because new shoreline space cannot simply be created within inland neighborhoods. Overcoming the severe isolation of coastal blue spaces means urban planning must transcend the immediate waterfront. Planners can effectively expand the daily living circles of inland residents by implementing pedestrian-priority crossings across coastal arterial roads and establishing continuous shaded green corridors. Direct public transit routes to waterfronts also help connect vulnerable populations to these geographically limited resources. Furthermore, environmental upgrading must always be accompanied by robust anti-displacement measures. Green investments frequently raise nearby land values and place immense pressure on lower-income renters (76). Improvement programs should therefore incorporate affordable housing protection and tenant consultation to prevent the transfer of public health benefits to wealthier demographic groups. A comprehensive monitoring system tracking pedestrian network continuity, maintenance response times, and displacement pressure is essential. Engaging older adults and migrant residents in participatory audits would ultimately make environmental access policy more precise, equitable, and accountable.

### 5.4 Limitations and future research

Several limitations should be acknowledged. The study estimated potential spatial access to health-supportive blue–green environments rather than direct health effects. Essential qualitative attributes encompassing vegetation density, biodiversity, safety, service capacity, and general cleanliness were excluded from this analysis although such factors significantly influence actual experiences. Following the concept of environmental affordances, the exposure index indicates opportunities for environmental contact (77). Actual effects may be weakened by crowding, noise, and poor maintenance, all of which are common across high-density cities. Researchers must incorporate crucial variables regarding water quality, scenic quality, extreme weather conditions, and site crowding into subsequent investigations. Future research should combine spatial exposure models with specific quality indicators, post-occupancy evaluation, environmental perception surveys, or qualitative place-based interviews to examine how residents actually use and benefit from blue–green spaces.

The operational definition of blue space also has limits. This study focused on publicly accessible coastal blue spaces because the coastal dimension is central to Shenzhen’s urban identity and planning structure. Moreover, inland rivers, reservoirs, wetlands, lakes, canals, and visual water exposure are generally embedded within urban parks and were captured in the green-space assessment. Conversely, non-park aquatic environments are frequently restricted for safety, flood control, or ecological management. Including these restricted waters would artificially inflate estimates of residents’ actual recreational access. Future research could distinguish usable, visible, and restricted blue spaces to refine blue-exposome assessment.

The Social Vulnerability Index relies on housing proxies including price, management fees, building age, and commercial structure. Strict data confidentiality at the local level in China prevented the inclusion of vital demographic dimensions such as income, education, employment, age, disability, migration status, and household characteristics. While residential metrics strongly reflect socioeconomic status in megacities, this proxy method inherently fails to capture the full spectrum of multidimensional vulnerability. Moreover, OpenStreetMap datasets occasionally exhibit spatial lags missing recent urban road expansions. Origin nodes directing journeys toward blue–green spaces were uniformly designated at residential street intersections reflecting enclosed community entrances. Enforcing this uniform configuration implies modeled travel durations for open neighborhoods might be overestimated. Pedestrian crossings were excluded from the spatial routing model because the foundational street dataset comprises single line features completely lacking physical width attributes. These combined routing and mapping limitations marginally compromise pedestrian accessibility estimations. Certain methodological limitations restrict the precision of the current accessibility modeling. Relying on a uniform walking speed inevitably overestimates spatial access for vulnerable demographic groups, particularly individuals with mobility impairments. Furthermore, micro scale environmental frictions remain unaccounted for within the spatial network dataset. Elements like steep terrain, traffic signal waiting times, and poor pavement conditions act as real world barriers that alter travel efficiency. Overcoming these generalized behavioral assumptions requires targeted analytical improvements. Subsequent research would benefit significantly from integrating continuous distance decay functions alongside demographic specific mobility parameters, which can substantially refine the evaluation of place-based wellbeing opportunities.

It is important to note that while the analytical algorithms employed in this research are well-established, the principal contribution of this study lies in their interdisciplinary synthesis and scalar refinement. By integrating these tools with a precise socioeconomic vulnerability index, the analysis moves beyond broad regional patterns to evaluate everyday environmental exposure. Specific neighborhoods facing a dual burden of severe vulnerability and limited access to natural health resources are thereby identified. Consequently, this integrated framework provides urban planners with actionable, place-based evidence for targeted micro-renewals, bridging the gap between theoretical environmental justice and practical, health-oriented urban design.

## 6 Conclusion

Taking Shenzhen as a coastal metropolis, this study examined residential inequality in potential access to public green spaces and publicly accessible coastal blue spaces. Utilizing 4,071 residential patches, we integrated a proxy-based social vulnerability index with walking-network accessibility, a 15-min living-circle threshold, and a capacity-weighted supply–demand model.

The results reveal that green-space exposure is spatially fragmented and disproportionately constrained for highly vulnerable populations. Coastal blue-space exposure exhibits even more acute concentration: the vast majority of residential lands face severe accessibility deficits, with high exposure strictly confined to a narrow coastal belt. Crucially, areas suffering from the “double burden” of high vulnerability and low exposure are predominantly concentrated in older, high-density, and peripheral neighborhoods. Consequently, the primary contribution of this study is a spatially explicit environmental-justice diagnosis, precisely identifying where access is restricted and how these limitations compound existing social vulnerabilities.

To address these disparities, planning responses must prioritize these identified high-vulnerability/low-exposure neighborhoods. Urban governance should transition from aggregate, macro-level ecological expansion toward targeted, neighborhood-scale interventions. By prioritizing micro-renewal, dismantling pedestrian barriers, and fostering inclusive spatial governance, planners can ensure that the health-supportive benefits of urban nature are equitably accessible to all.

## Data Availability

The original contributions presented in the study are included in the article/Supplementary material, further inquiries can be directed to the corresponding author.
