# Supplementary material for: Unequal access to restorative coastal blue–green places: social vulnerability and place-based well-being opportunities in a coastal metropolis
Source: Front Public Health. 2026 Oct 7;14:1917150. doi: 10.3389/fpubh.2026.1917150 (PMC13683656; doi:10.3389/fpubh.2026.1917150)
Supplement: Supplementary file 1 [file Data_Sheet_1.pdf]

Supplementary materials for

Unequal Access to Restorative Coastal Blue–Green Places: Social Vulnerability and Place-Based Well-Being Opportunities in a Coastal Metropolis

Baijing Liu[1], Xinping Yu[1], Lu Yu[2,3], Hongzhen Zheng[4], Guannan Li[5*]

[1]School of Resources and Environmental Engineering, Ludong University, Yantai, Shandong, China

[2]Jinan Land Reserve Center, Jinan, Shandong, China

[3]School of Water Conservancy and Environment, University of Jinan, Jinan, Shandong, China

[4]Zhanhua District Natural Resources Bureau of Binzhou City, Binzhou, Shandong, China

[5]Ulsan Ship and Ocean College, Ludong University, Yantai, Shandong, China

Table S1 Pairwise Spearman rank correlations among sub-indicators of the Social Vulnerability Index.

		**Housing market**	**Property management**	**Dwelling age**	**Commercial-service structure**
**Housing market**	$\|\rho\|$	1	0.111	0.081	0.036
	P	—	<0.001	<0.001	<0.05
**Property management**	$\|\rho\|$	0.111	1	0.333	0.234
	P	<0.001	—	<0.001	<0.001
**Dwelling age**	$\|\rho\|$	0.081	0.333	1	0.146
	P	<0.001	<0.001	—	<0.001
**Commercial-service structure**	$\|\rho\|$	0.036	0.234	0.146	1
	P	<0.05	<0.001	<0.001	—

Note: Values are the absolute Spearman's rank correlation coefficients ($|\rho|$). $|\rho|\geq 0.80$ was considered indicative of potentially substantial pairwise redundancy.

Table S2 Auxiliary-regression-based multicollinearity diagnostics (R^2, tolerance and VIF) for sub-indicators of the Social Vulnerability Index.

	**Auxiliary-regression (R^2)**	**Tolerance**	**VIF**
**Housing market**	0.005	0.995	1.005
**Property management**	0.106	0.894	1.119
**Dwelling age**	0.087	0.913	1.095
**Commercial-service structure**	0.057	0.943	1.060

Note: Each indicator was regressed on the remaining three indicators. VIF values greater than 5 were considered indicative of potentially problematic multicollinearity.

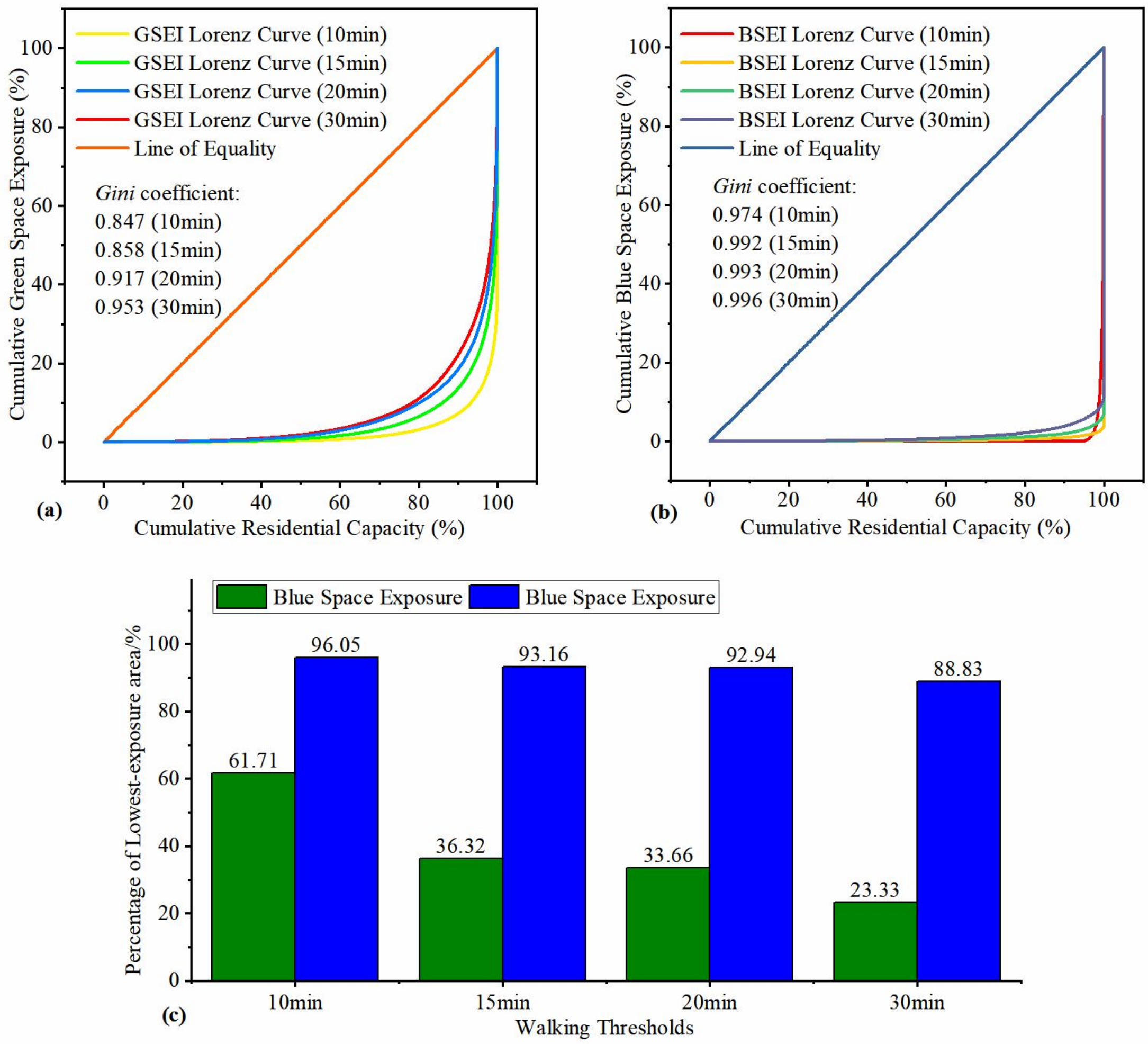

Figure S1. Sensitivity analysis of spatial accessibility outcomes under alternative pedestrian catchment thresholds. (a) Lorenz curves and Gini coefficients for green-space exposure; (b) Lorenz curves and Gini coefficients for blue-space exposure; and (c) percentages of areas with the lowest green- and blue-space exposure under 10-, 15-, 20-, and 30-minute walking thresholds. Abbreviations: GSEI—Green Space Exposure Index, BSEI—Blue Space Exposure Index.
